# Bacteriocins as Potential Therapeutic Approaches in the Treatment of Various Cancers: A Review of In Vitro Studies

**DOI:** 10.3390/cancers14194758

**Published:** 2022-09-29

**Authors:** Arnold Marshall Molujin, Sahar Abbasiliasi, Armania Nurdin, Ping-Chin Lee, Jualang Azlan Gansau, Roslina Jawan

**Affiliations:** 1 Biotechnology Programme, Faculty of Science and Natural Resources, Universiti Malaysia Sabah, Jalan UMS, Kota Kinabalu 88400, Sabah, Malaysia; 2 Halal Products Research Institute, Universiti Putra Malaysia, UPM Serdang 43400, Selangor, Malaysia; 3 Department of Biomedical Sciences, Faculty of Medicine and Health Sciences, Universiti Putra Malaysia, UPM Serdang 43400, Selangor, Malaysia; 4 Biotechnology Research Institute, Universiti Malaysia Sabah, Jalan UMS, Kota Kinabalu 88400, Sabah, Malaysia

**Keywords:** lactic acid bacteria, bacteriocin, cancer, anticancer, new treatment

## Abstract

**Simple Summary:**

Current cancer treatment strategies such as surgery, chemotherapy, and radiotherapy, have significant drawbacks. There is a need for a breakthrough approach to cancer treatment. Bacteriocin, an antimicrobial peptide, has shown several anticancer properties in vitro. Therefore, this article reviews the effect of bacteriocin on cancer cells and how bacteriocins affect cancer cells in vitro. This article aims to promote additional bacteriocin research, particularly in vivo studies, to fully understand the potential of bacteriocin as a cancer treatment agent.

**Abstract:**

Cancer is regarded as one of the most common and leading causes of death. Despite the availability of conventional treatments against cancer cells, current treatments are not the optimal treatment for cancer as they possess the possibility of causing various unwanted side effects to the body. As a result, this prompts a search for an alternative treatment without exhibiting any additional side effects. One of the promising novel therapeutic candidates against cancer is an antimicrobial peptide produced by bacteria called bacteriocin. It is a non-toxic peptide that is reported to exhibit potency against cancer cell lines. Experimental studies have outlined the therapeutic potential of bacteriocin against various cancer cell lines. In this review article, the paper focuses on the various bacteriocins and their cytotoxic effects, mode of action and efficacies as therapeutic agents against various cancer cell lines.

## 1. Introduction

An estimated 19.3 million new cases of cancer and 10 million cancer-related deaths worldwide were reported in 2020 [1]. Cancer is a complex systemic disease with different variants and is one of the major causes of death for non-communicable diseases worldwide [2,3]. The cells that develop into cancer are altered cells that fail to respond to the usual controlling signals and their growth becomes unregulated [4,5].

Ideally, cancer treatment involves completely removing both tumours and metastases. The current cancer treatments available include surgery, chemotherapy, and radiotherapy. However, each treatment has drawbacks, either requiring additional treatment or lacking target specificity resulting in additional side effects on the patient. For instance, chemotherapy and radiotherapy have low specificity toward cancer cells, eliminating healthy and diseased cells with detrimental side effects [6]. In addition, these treatments cannot completely remove tumours and metastases [7,8,9].

The limitations of the current cancer treatments call for alternative interventions since cancer cells continue to evolve and build resistance against existing chemotherapeutic agents [10]. There is a dire need for new molecules with higher selectivity and specificity against cancer cells. Microorganisms, particularly bacteria, possess a broad range of proteins and peptides with antitumoral properties such as toxins, immunotoxins, enzymes, and bacteriocins that offer potential bioremediation. For instance, bacteriocins possess potent biological properties due to their unique structural features. Their functions were initially thought to be limited to inhibiting bacterial growth and are now expanded to suppressing various cancer cell lines [7,11,12]. Therefore, this review highlights bacteriocin as a potential anticancer agent and the mechanisms involved in inhibiting the growth of cancer cells.

## 2. Cancer

Tumour clonality, or the growth of tumours from single cells that start to multiply abnormally, is one of the essential characteristics of cancer. However, the clonal origin of tumours does not always mean that the first progenitor cell that gives rise to a tumour has always possessed all the traits of a cancer cell. Contrarily, the emergence of cancer is a multi-step process in which malignant cells gradually develop through a series of mutations [13]. When normal cells accumulate genetic mutation over time, their normal functions of cell proliferation become dysregulated, thus producing malignant cells, leading to a more proliferative, invasive, and metastatic disease [4,14]. Any tissue in the body can develop into cancer, although some sites are more prone than others. The lungs, breasts, prostate, GI tract, and skin are the most common. Although almost all tumours start out as a single mutant cell, they eventually become heterogeneous, meaning the cancer cells express different markers, proliferate more, and become more differentiated than normal cells [5,15].

### 2.1. Diagnosis of Cancers

Depending on the location, size of the tumour, and type of cancer, different symptoms may be present. In some cancer, symptoms may develop when the cancer is in the early stages, for example, even a small tumour of the brain can cause pressure to develop. Other tumours, such as colorectal cancer may not show symptoms until it reaches the advanced stages [5,16].

Diagnostic imaging may be used to assess patients exhibiting cancer symptoms in order to locate the potential tumour. X-rays, computed tomography (CT), magnetic resonance imaging (MRI), positron emission tomography (PET), and ultrasound are all examples of diagnostic imaging [17]. Another method of diagnosing a cancer patient is by performing a biopsy, which involves taking a sample of the patient’s solid tumours. Endoscopy is a technique for taking biopsies; it enables one to pinpoint the tumour’s location and remove a section of it from the large intestine for histological analysis [18]. Histological analysis visually examines the regularities of cell shapes and tissue distributions. This will make it possible to assess the level of malignancy and also identify whether the tissue regions are cancerous [19].

A staging system for cancer is used to document the severity of the tumour experienced by the patients and the TNM staging, where T records the distance the tumour had grown from the original location, N describes whether the tumour had spread to the local lymph nodes, and M describes whether the tumour has metastasized [5].

### 2.2. Treatment Options for Cancer

Once a patient is diagnosed with cancer, the ideal management process will involve the complete removal of the tumour and metastases. However, the treatment option depends on the stages and location of the cancer [8,16].

#### 2.2.1. Surgical Treatment 

Surgery can eliminate the early stages of cancer while also halting the development and spread of the cancer cells. Surgery, however, is only an option if the cancer cells have not spread, and some surgeries would need further therapy, such as chemotherapy, to entirely eradicate the cancer cells. Oncologic surgery’s primary goals are to achieve local control, avoid locoregional recurrence, increase patient survival rates, relieve symptoms, and improve patients’ quality of life [16,20,21].

Following surgery, complications may arise; these complications can range in severity from minor to severe, depending on the cancer type and surgical technique. After surgery, infections and bleeding are potential side effects, and certain complications may even be fatal. For instance, axillary web syndrome (AWS) may develop following breast cancer surgery, which influences the patient’s physical appearance [21,22]. While patients with colorectal cancer who have total mesorectal excision (TME) run the risk of anastomosis dehiscence (AD) and severe anorectal dysfunction. The digestive system’s airtightness is compromised by AD, which enables the interior of the digestive tract to communicate with the extraluminal area. As a result, clinical signs and symptoms may appear, which may ultimately lead to the patient’s death [23,24]. Surgery can also lead to the proliferation of metastatic cancer cells due to the decrease of inhibitor levels when the primary tumour is removed. The ability of the primary tumour to slow down the growth of metastatic foci is known as concomitant tumour resistance. Primary tumours secrete both proangiogenic factors (inducers) and inhibitors of angiogenesis. The inducers in the primary tumour’s microenvironment counter the inhibitors’ actions, which is essential for the progression of tumour growth. In circulation, the levels of the more stable inhibitors produce a systemic antiangiogenic environment that prevents small distant micrometastases from developing and inducing neovascularization, while the levels of the more labile inducers fall off quickly. These micrometastases, therefore, remain tiny and inactive. When the primary tumour is removed, inhibitor levels drop, and the previously dormant metastases begin to grow [25].

#### 2.2.2. Chemotherapy

Chemotherapy aims to reduce the volume of cancerous tissue, either by increasing cell loss or reducing the number of tumour cells produced. Cytotoxic drugs interfere with cell division, resulting in declining new cell production, and preventing invasion and metastasis from happening [26,27]. Therapeutic failure can be due to either genetic changes of the cancer cells or induced by drug treatment. A huge challenge in the fight against cancer continues to be the resistance mechanisms of cancer cells. Each year, the Food and Drug Administration (FDA) approves new chemotherapeutic medications to battle the rising resistance of cancer cells. Lurbinectedin, one of the recent chemotherapeutic drugs, was approved by the FDA on 15 June 2020 for patients with metastatic small cell lung cancer (SCLC) [28,29].

Every known cytotoxic drug has harmful side effects. Different drugs vary in toxicity but are identically detrimental due to the inability to distinguish between normal and malignant cells. When a cytotoxic drug is introduced into the body, the chemical compound attacks the proliferating tumour cells and healthy cells [26]. Consequently, targeted therapies are gaining popularity since the treatment can specifically target cancer cells by inhibiting cell proliferation, differentiation, and migration. Small molecules with a molecular weight of <900 Da penetrate the cells and inactivate selected enzymes, interfering with tumour cell growth and triggering cell apoptosis [30].

Generally, targeted therapy increases the survival rate compared to conventional chemotherapy. However, this treatment has several drawbacks, including the high running cost that increases the financial burden for patients, especially with the combination of two or more targeted agents. Furthermore, crossover and bypass of mechanisms occur between pathways due to the acquired resistance from exposure to these targeted treatments, and the efficacy of this treatment varies between individuals [8].

#### 2.2.3. Radiotherapy

Radiotherapy exposes cancer cells to high-energy radioactive particles, causing DNA damage and cell death [16,31]. The aim of radiotherapy is to treat all macroscopic tumours in areas at risk of local recurrence and regional lymphatics by delivering as much dose to the tumour whilst sparing normal tissues. There are two methods for administering radiation to the cancer [32,33]. Radiation therapy is delivered on a fractionated dosage regime which differs between different types of cancer based on the differing radiobiological properties of cancer and various normal tissues. For a typical radiation therapy regime, the daily fractions dosage is around 1.5 to 3 Gy that spreads out over several weeks [33]. The genes responsible for cell proliferation and cellular repair will undergo mutation, and the rate of chromosomal aberrations will depend on the radiation exposure [31].

Radiotherapy toxicity towards normal cells depends on the radiation technique and volume of normal tissues irradiated, including perforation, obstruction, strictures, malabsorption, increased bowel frequency, incontinence, infertility, erectile dysfunction, and delayed delivery wound healing [32]. Furthermore, a major downside of radiotherapy is the risk of secondary malignancies. Despite the efficacy of ionising radiation in destroying cancerous tissues, the incidence of second malignant neoplasm can develop from cancer cells that survived the treatment since ionising agents are also carcinogenic and change the patient’s chromosomal DNA [31].

## 3. Potential Treatment of Cancer

Factors associated with cancer are diets, lifestyle, genetics, oncogenic infections, and the variability of the microbiome, especially in the gut. The gut microbiota plays an important role in maintaining gut health; thus, any changes to the gut microbiome may result in the emergence and development of cancer. A bacteria group that plays a significant role in the gut microbiota are probiotics, live microorganisms that provide health benefits to their host. Having adequate probiotics in the gut can potentially prevent cancer development and progression. Lactic acid bacteria (LAB) is the most common group of probiotics in the human gut [34,35].

A plethora of antimicrobial peptides (AMPs) is produced by LAB, including bacteriocins. Bacteriocins are naturally found in food promoting bacterial growth, such as fermented food. Many bacteriocins have been characterised at the biochemical and genetic levels and tested as food biopreservatives against pathogenic bacteria and spoilage [36]. Bacteriocins produced by LAB are generally regarded as safe (GRAS) since LABs are also classified as GRAS [37].

Bacteriocins are commonly used as food preservatives owing to their antibacterial properties. While bacteriocins are useful in preventing bacterial growth in food, their anticancer activities have been explored only recently. Nisin is the most well-studied and widely used as a food preservative. Despite its sterling reputation as an antibacterial agent, knowledge regarding its ability to inhibit cell growth is still in its infancy. Nisin perforation of the cancer cell membrane may significantly induce apoptosis, and thus, hinder cell proliferation. Additionally, the pores may allow the influx of calcium ions into the cell which activates apoptosis. Calcium ions activate the apoptosis pathway by altering the activation of cell surface death receptors and caspases which allows apoptosomes to react [38].

## 4. Bacteriocin

Bacteriocins are a group of ribosomally-synthesised cationic bacterial peptides secreted by Gram-positive bacteria, namely probiotics, and are classified as bacterial antimicrobial peptides. Bacteriocins produced by lactic acid bacteria (LAB) have gained the interest of researchers because of their potential as a natural food preservative and therapeutic agent. Furthermore, bacteriocins exhibit antibacterial properties with narrow to broad-spectrum activities [39,40]. In addition, LAB is widely used in bacteriocins production because they carry genes associated with transferable elements such as conjugative transposons or plasmids that can be transformed into other bacterial strains in a non-recombinant way [41,42].

The genes for bacteriocin biosynthesis are clustered on plasmids, chromosomes, and transposons. Bacteriocins are synthesised as biologically inactive peptides with an N-terminal leader peptide attached to the C-terminal propeptide. The leader peptide has several functions, including being the recognition site that allows the prepeptide to mature and transport protein, protecting the bacteriocin producer from harming itself by keeping the bacteriocin in an inactive state and interacting with the propeptide domain to ensure that the peptide is in the correct conformation for enzyme–substrate interaction of the modification machinery [39,42].

Bacteriocins, especially from LAB, are actively studied for food and medical application. Bacteriocins are crucial in promoting the colonisation of LAB in the host’s intestine, eliminating pathogenic bacteria, and acting as a signalling peptide that regulates the host’s physiological function, especially the immune system [43]. Bacteriocins possess unique and valuable traits such as tolerance toward high thermal stress and remain viable over a broad pH range. Furthermore, bacteriocins are colourless, odourless, and tasteless, further enhancing their potential application. Since the beginning of the bacteriocins utilisation, there have been no reports of bacteria resistance, possibly due to the fast-acting mechanism which prevented the ability of target cells to develop resistance even at low concentrations. Furthermore, bacteriocins are vulnerable in the environment and are easily degraded due to their proteinaceous nature, thus, reducing the opportunity of target cells to interact and form resistance [39]. Thus, bacteriocins are sustainable for the food industry and medical field.

### 4.1. Classes of Bacteriocins

Bacteriocins are classified into three groups based on their mechanism of biosynthesis and biological activity. The parameters considered for bacteriocins classification are host producer, intrinsic function, molecular weight, physicochemical properties, and amino acid sequence.

#### 4.1.1. Class I: Ribosomally-Produced and Posttranslationally-Modified Peptides (RiPPs)

The RiPPs are also known as lantibiotics. They are small, heat-stable peptides (less than 10 kDa) that have been subjected to enzymatic modification post-translation. The enzymatic modification involves structural changes such as the addition of heterocycles, head-to-tail cyclisation and glycosylation, giving rise to uncommon amino acids such as lanthionine (Lan), methyllanthionine (MeLan), dehydroalanine (Dha), dehydrobutyrine (Dhb), and D-alanine (D-Ala). Furthermore, lantibiotics contain leader peptides which act as a site for enzyme recognition, transport and maintaining the inactive state of the peptide [42,44].

Lantibiotics are further classified into two different groups based on the charges. Type A lantibiotics are 2–4 kDa long, positively charged, screw-shaped, flexible molecules such as nisin and lacticin 3147. They perforate the cell membrane of the target organisms, leading to the depolarisation of the cytoplasmic membrane. Meanwhile, type B lantibiotics are 2–3 kDa long, with no net charge or net negative charge. These structurally-globular lantibiotics interfere with cellular enzymatic reactions by disrupting cell wall synthesis. An example of type B lantibiotic is mersacidin secreted by *Bacillus* spp. [39,44,45,46].

#### 4.1.2. Class II: Thermostable Unmodified Bacteriocins

Thermostable, unmodified bacteriocins are small (<10 kDa), heat-stable, that may undergo the formation of a disulphide bridge. No enzymes are required for the maturation of these molecules besides the removal of leader peptides and the formation of a conserved N-terminal disulphide bridge. The amphiphilic, helical structure of these bacteriocins allows them to proliferate the target cell membrane, leading to depolarisation and cell death [42,44].

These antimicrobial peptides are subdivided into three subclasses: IIa, IIb, and IIc. Subclass IIa bacteriocins have a distinct, conserved sequence located in the N-terminal consensus sequence, resulting in their high potency, particularly against *Listeria monocytogenes*. Examples of subclass IIa bacteriocins are pediocin PA-1 and sakacin-A. On the other hand, subclass IIb bacteriocins consist of two different peptides that work synergistically to generate an antimicrobial effect. Examples of subclass IIb bacteriocins are lactacin F and lactococcin G [39,45].

The IIc subclass comprises circular-structured bacteriocins carrying two transmembrane segments that facilitate pore formation on target cells. Bacteriocins in this class lack the N-terminal leader peptide; instead, the N-termini and C-termini are covalently linked, forming a stable and circular structure. Examples of IIc bacteriocins are gassericin A, circularin A, and carnocyclin A. Nevertheless, some studies suggested circular bacteriocins should be classified under a separate class [39,44,45,46].

#### 4.1.3. Class III: Thermolabile Unmodified Bacteriocins

Thermolabile unmodified bacteriocins are heat-labile with a high molecular weight (>10 kDa). These antimicrobial peptides are subdivided into two groups: bacteriolysins and non-bacteriolytic. Bacteriolysins exhibit antimicrobial activity by cleaving the peptidoglycan cross-links of the target cells’ cell walls. Examples of bacteriolysins are helveticin V-1829 secreted from *Lactobacillus helveticus*, and lysostaphin secreted from *Staphylococcus simulans*. Examples of non-bacteriolytic are colicins, megacins secreted by *Bacillus megaterium*, klebicin secreted by *Klebsiella pneumonia*, and enterolysin secreted by *E. faecalis* [39,44,45,46].

### 4.2. Bacteriocins for Anticancer Treatment

The conventional anticancer treatment for cancer is endoscopy, chemotherapy, radiotherapy, and surgery. Nonetheless, these treatment options have drawbacks. For instance, chemotherapy affects cancer cells and normal cells, resulting in chemoresistance development [9,10]. Therefore, a new form of treatment is required with better precision and side effects.

Studies have been conducted to identify the anticancer properties of bacteriocins. Bacteria produce metabolites, including antimicrobial peptides (AMPs), to overcome the competition against other invading bacteria and one of the AMPs is the bacteriocins which were initially thought to inhibit the growth of other related bacterial strains. Since then, recent studies have indicated the presence of broad-spectrum and selective antimicrobial activity against strains that are distantly related. Moreover, bacteriocins have been shown to inhibit the growth of various cancer cell lines [11].

Bacteriocins anticancer properties are profound due to their ability to differentiate cancer cells from non-cancer cells. Cancer cell membrane surfaces are negatively charged, whereas non-cancer cells are neutral. This characteristic can be attributed to anionic phosphatidylserine, gangliosides, heparin sulphates and *O*-glycosylated mucins in cancer cells. As for non-cancer cells, the surface membrane consists of neutral phospholipids (i.e., sphingomyelins and phosphatidyl choline), while the inner surface contains amino phospholipids [10]. Most bacteriocins with anticancer properties are cationic and amphiphilic with a high affinity towards the negative surface charge of the cancer cells, thus, allowing them to selectively target the cancer cells without affecting healthy cells. In addition, the cancer cell membrane has a high fluidity which results in the destabilisation of the cell membrane. The high number of microvilli on the surface of the cancer cells further assists in the binding and uptake of bacteriocins. Ultimately, the cancer cells’ binding and uptake of bacteriocins lead to their demise, mediated via cell membrane lysis [10,11].

Bacteriocins induce apoptosis in cancer cells. A study by Ahmadi et al. (2017) demonstrated how nisin affects the apoptotic pathway of colorectal cancer (CRC) cells. Nisin regulated the cancer cell signalling pathway by promoting apoptosis in cancer cells via the intrinsic pathway, mediated by mitochondria and the B-cell lymphoma 2 (BCL-2) family (comprising pro-apoptotic proteins such as BCL-2-associated X protein (BAX) and anti-apoptotic proteins such as BCL-2). Precisely, nisin causes an imbalance in the expression of BAX/BCL-2 expression ratio and a higher expression of the pro-apoptotic proteins at the messenger ribonucleic acid (mRNA) and protein level of the cancer cells. As a result, the apoptotic index increases, which causes apoptosis to occur [34].

According to Joo et al. (2012), cancer cells are naturally resistant to apoptosis. The apoptosis mechanisms began with the release of cytochrome c by the mitochondria, catalysing the endoplasmic reticulum to release calcium. These two components are essential in apoptosome formation, activating caspases and nucleases that will cleave substrates and DNA to propagate apoptosis. Calcium is an important ion, especially in mediating apoptosis by altering the activation of cell surface death receptors and caspases [38].

Table 1 shows studies regarding bacteriocins that possess anticancer properties. The bacteriocins were isolated from various bacteria sources, most of them from lactic acid bacteria. Furthermore, these microbial peptides affect other cancer cell lines besides colon cancer. Bacteriocins’ ability to inhibit cancer cell proliferation was also proven through various assays.

**Figure 1:**
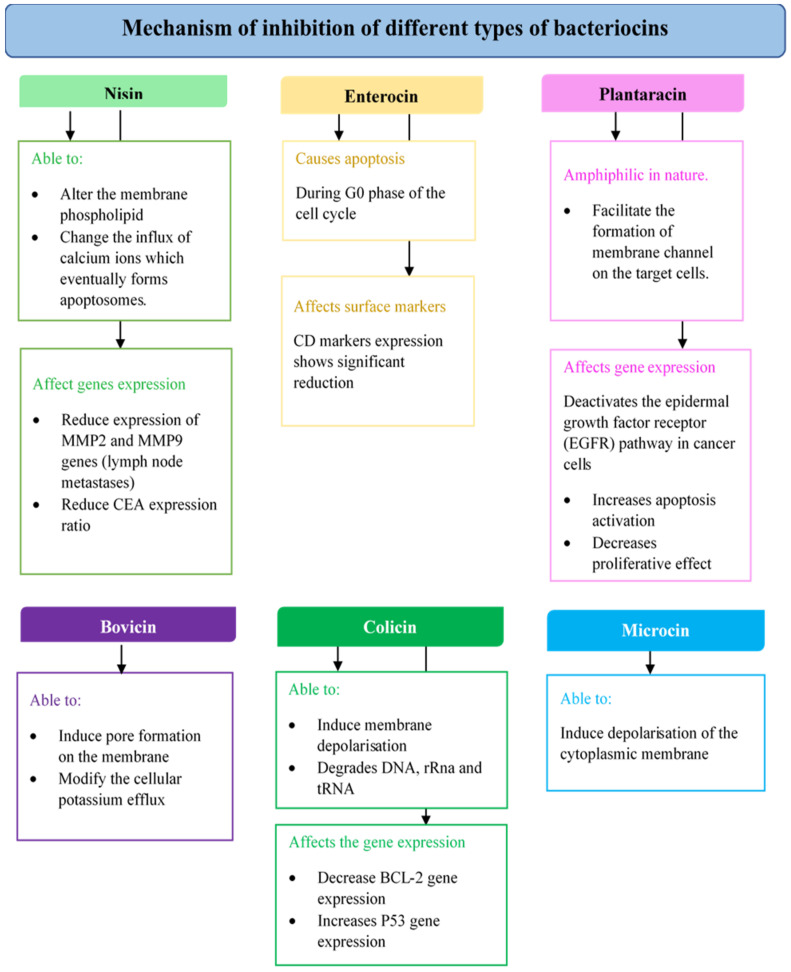
Mechanism of action of different bacteriocins on cancer cells.

Figure 1 shows a summary of the mechanism of action of various types of bacteriocins against cancer cell lines. Different bacteriocins are shown to have different inhibitory effects on cancer cells, with some even affecting the cancer cell gene expression mechanism.

#### 4.2.1. Nisin

Nisin is a low molecular weight lantibiotic bacteriocin (~3 kDa) produced by *Lactococcus lactis* subsp. *lactis*. This microbial peptide exhibits antibacterial activity against different bacteria, including pathogenic strains. In addition, nisin shares similarities with other pore-forming AMPs, such as having a positive net charge and amphipathicity. The ability to perforate pathogenic strains and exhibit low toxicity towards other cells makes nisin an ideal molecule for the food industry. Furthermore, reports have suggested nisin as an anticancer agent [7].

A study by Joo et al. (2012) identified the antitumor potential of nisin on head and neck squamous cell carcinoma (HNSCC) cells. When the HNSCC cells were treated with nisin, the apoptosis rate increased, and the cell proliferation rate decreased. Moreover, nisin can alter the membrane phospholipids and the influx of calcium ions into the cells. The calcium influx would lead to apoptosome formation and, eventually, apoptosis. In addition, nisin also affects genes that are involved in the cell physiology which includes apoptosis, cell cycle pathways, membrane physiology, ion transport, energy and nutrient pathways, protein binding, and signal transduction pathways [38].

Norouzi et al. (2018) tested nisin for anticancer properties against CRC cells (LS180, SW48, HT29 and Caco-2 cell lines). Furthermore, nisin treatment reduced the expression matrix metalloproteinase (MMPs) and carcinoembryonic antigen (CEA) genes in HT-29, Caco-2, LS180 and SW48 cells compared to untreated cancer cells. The MMPs and CEA are important molecular biomarkers for detecting colon cancer metastasis [2]. The MMPs play an important role in cancer development and regulate signalling pathways. Nisin reduced the expression of MMP2 and MMP9 while increasing the expression of inhibitors. MMP-2 and MMP-9 are associated with lymph node metastasis. Nisin also reduced the ratio of CEA expression in the colon cancer cell lines by approximately three-fold. The ELISA assay identified CEA and CEAM6 as biomarkers and a transport mechanism. In addition, Norouzi et al. (2018) found that nisin at different concentrations exhibited significant reductions in tumour volumes.

#### 4.2.2. Enterocin

Enterocin is a broad-spectrum bacteriocin known to be diverse in the form of different classes, even when it originates from the same host bacterial species. Himeno et al. (2015) reported that the *E. faecium* NKR-5-3 strain produces five types of enterocins from different fractions, namely NKR-5-3A, B, C, D, and Z. Enterocin NKR-5-3A and Z belong to class IIb, NKR-5-3c in class IIa, while NKR-5-3D is in class IId and the weakest in antibacterial activity [54].

Al-Madboly et al. (2020) studied the effect of enterocin LNS18 against HepG2 cell line. There was a significant increase in the cell count during the G_0_ phase compared to untreated cells, indicating the increase in apoptosis in the presence of enterocin LNS18. Furthermore, the addition of enterocin LNS18 significantly reduced CD surface markers’ expression in HepG2 cells [10].

#### 4.2.3. Plantaricin 

Plantaricin is produced by different strains of *Lactobacillus plantarum* with a low molecular weight (~2 kDa) and is classified as class IIb bacteriocins. The amphiphilic nature of the plantaricin bacteriocin facilitates the formation of membrane channels. Plantaricin also has a high affinity toward negatively-charged membranes and interacts strongly with glycolate membrane proteins [7].

De Giani et al. (2019) isolated plantaricin P1053 from *Lactobacillus plantarum* obtained from human faeces. This microbial peptide has a molecular weight of 1053 Da and exhibited a broad-spectrum activity against Gram-negative and Gram-positive bacteria. Plantaricin P1053 was tested on colon CCD 841, a human intestinal epithelial cell, and the cell viability improved by approximately 20%. Furthermore, plantaricin P1053 demonstrated anticancer activity against colon cancer cells, E705 cells, with a significant inhibitory effect of nearly 30%. It was also discovered that the bacteriocin activates the epidermal growth factor receptor (EGFR) pathway in the CCD 841 healthy cell line, increasing anti-apoptotic and pro-proliferative effects. Meanwhile, plantaricin 1053 does not activate the EGFR pathway on E705 cells, thus preventing the increase in cell viability [48].

#### 4.2.4. Pediocin

*Pediococcus* bacteria produces pediocin, small plasmid-encoded cationic AMPs larger than 5 kDa, with high stability at various temperatures and pH. Pediocins structure contains two regions which are: an N-terminal region that mediates the binding of pediocins to the target cell membrane; and the C-terminal region that enables pediocin to penetrate the target cell membrane hydrophobic region, creating leakage through the membrane [7,55,56].

Villarante et al. (2011) extracted pediocin from *Pediococcus acidilactici* K2a2-3 isolated from the intestines of Philippine water buffalo. They demonstrated the bacteriocin cytotoxicity activity against human colon adenocarcinoma (HT29) and human cervical carcinoma (HeLa) cell lines. There was no significant difference between dialysed and undialysed pediocin in the growth of HT29 cells. However, for HeLa cells, there is a significant difference between dialysed and undialysed pediocin K2a2-3. According to Villarante et al. (2011), the loss of cytotoxic activity of dialysed pediocin K2a2-3 towards HeLa cells is due to the dilution process during dialysis [49].

#### 4.2.5. Bovicin

Bovicin is a low molecular weight lantibiotic bacteriocin (2.4 kDa) produced by *Streptococcus bovis* HC5. This microbial peptide is a class I bacteriocin, stable at high temperature and low pH and exhibits a broad-spectrum antimicrobial activity. Bovicin has a similar structure and function as nisin, causing pore formation on the membrane and modifying the cellular potassium efflux of the cells [7,45].

A study by Paiva et al. (2012) found that despite sharing the same membrane target, bovicin HC5 perforating rate is different from nisin; the latter is more effective than the former due to the differences in size and mechanism of pore formation [47].

#### 4.2.6. Colicins 

Colicins are high molecular mass bacteriocins (27–80 kDa) originating from *Escherichia coli* (*E. coli*) and some species of *Enterobacteriaceae*, rich in diversity (E1-3, K, A, L, B, Ia, Ib, V, D, and M). Colicins bind to the outer membrane of the integral membrane protein receptors, transporting colicin to the inner membrane, which then induces membrane depolarisation and degrades the DNA, ribosomal RNA (rRNA), and transfer RNA (tRNA) [2,7]. Meanwhile, Taherikalani and Ghafourian (2021) identified that colicin E7 impacted HT29 colon cancer cell lines by decreasing *BCL-2* gene expression and increasing *P53* gene expression [4].

#### 4.2.7. Microcins

Microcins are often produced from the Enterobacteriaceae family, having a molecular mass of up to 10 kDa and exhibit antimicrobial activity against pathogenic bacteria such as *Salmonella*, *Enterobacter*, *Klebsiella*, *Escherichia*, and *Citrobacter*. These bacteriocins are classified into two classes based on their sizes: class I (<5 kDa) and class II are microcins (5–10 kDa). Microcins induce the depolarisation of the cytoplasmic membrane to reach a specific molecular target by taking advantage of the receptors of the cells, whereby the interaction with the receptor allows the microcin to translocate into the inner membrane protein complex [7].

Varas et al. (2020) reported the effectiveness of purified microcin E492 in CRC cell lines (HT29 and SW620). Microcin E492 has a molecular mass of approximately 8 kDa and is classified as a class II bacteriocin. The microcin E492 treatment significantly reduced the cell viabilities of HT29 (~20%) and SW620 (~10%), indicating a stronger cytotoxic efficacy in the former than the latter in vitro [50].

### 4.3. Bacteriocins In Vivo Study

A study by Joo et al. (2012) used a floor-of-mouth mouse model to study the effect of bacteriocin on oral cancer. Mouse mouth floors were submucosally injected with HNSCC cells. To determine if preloading with nisin would be effective, a nisin pretreatment group was used. Nisin administration (200 mg/kg per day) was started 3 weeks prior to tumour cell injections and continued for 3 additional weeks after. After the initial tumour cell injections (or as soon as tumour cell development was confirmed and palpable), Nisin (200 mg/kg per day) was once again given for 6 weeks. Rats given nisin doses of around 80 mg/kg did not show any negative effects, while mice given a 150 mg/kg dose of nisin over the period of three weeks showed normal weight gain and organ histology without any negative consequences. When compared to controls, mice treated with nisin showed statistically significant lower tumour volumes. Nisin preloading decreases the tumour volume, while nisin treatment has no effect on the histological morphology of the liver or kidneys [38].

A study by Varas et al. (2020) used the zebrafish model to study the effect of bacteriocin on human colorectal cancer. Zebrafish larvae were given transplanted with human cancer cells SW620. These cells were used to create a zebrafish xenograft model, which was used to assess the anticancer activity of microcin (MccE492) in vivo. Results showed that intratumor injection of this peptide dramatically decreased the tumour cell mass. A few days after transplantation, it was discovered that SW620 cells, which had been shown to be highly invasive in in vitro experiments, had spread widely in zebrafish larvae. The SW620 line was used because it was extremely invasive and frequently causes secondary cancers in its high number of individual zebrafish. The zebrafish xenograft model was used to assess primary patient-derived biopsy specimens, which are frequently challenging to culture in vitro. The model can be used to separate cancer cells and to produce xenografts in zebrafish, which are ready for testing the response of the cells to various anticancer drugs within a few days. The evidence suggests that bacteriocin is a novel antitumorigenic bacterial molecule with the advantages of being tiny, stable, and protease- and harsh-condition resistant, including boiling. One of the primary prerequisites for a molecule with a potential pharmacological application is stability. Since it is created by bacteria, there is also a chance for direct delivery via a probiotic infection used for therapeutic purposes [50]. There is currently insufficient study on in vivo tests for bacteriocins, particularly on clinical samples. Animal studies are the main focus of most recent experimental studies.

## 5. Complications Regarding Bacteriocins in Medical Applications

Despite the advantages that bacteriocins offer (small size, biocompatibility, biodegradability, and non-immunogenic), there are several limitations to their medical application. Issues relating to bacteriocin’s stability, solubility, mass production, and purification hampers their utilisation in the medical setting. Currently, medical peptides are manufactured via solid or liquid phase synthesis. The complex nature of bacteriocin peptides and their posttranslational modification requirement would be costly for large-scale production [46].

Another issue is the bacteriocin administration route for patients. Oral administration of bacteriocin is susceptible to degradation from enzymes or pH in the gastrointestinal tract. Further research on the pharmacokinetics of bacteriocin, which includes the intestinal absorption, bioavailability, distribution, half-life, and renal clearance of bacteriocins in the body, is required to fully understand how bacteriocin would reach the site of action and how the bacteriocin would be expelled from the body. Bacteriocins also have a much lower half-life than antibiotics due to their sensitivity to proteases in vivo [57].

Like drugs and antibiotics, bacteriocin resistance can develop with rampant use by degrading bacteriocin and adapting the cell membrane and growth condition to exist in the presence of bacteriocins. In addition, bacteriocin resistance may also be passed chromosomally to other cells, mimicking the transfer of antibiotic resistance genes to other cells [58].

## 6. Conclusions

Cancer is a leading cause of non-communicable diseases and mortality worldwide. The current conventional treatments are plagued with problems such as drug resistance of cancer cells and the lack of specificity in targeting cancer cells without affecting healthy cells. Researchers have begun exploring the potential of microorganisms such as bacteria due to the plethora of potentially beneficial molecules in targeting cancer cells, including bacteriocins. Bacteriocin is a promising anticancer agent by offering specificity against target cells without affecting healthy cells. Various bacteriocins that exhibit anticancer properties and the mechanisms of action were discussed in this review. However, there is a limitation in in vivo studies on the anticancer effects of bacteriocins on cancer cell lines. Hence, a more in-depth investigation is essential for elucidating the characteristics of bacteriocins as anticancer agents in vivo and in clinical settings. Nonetheless, previous in vitro and in vivo studies showed that bacteriocins are promising as anticancer agents for cancer treatment. Further research will help uncover novel bacteriocins with anticancer properties that can complement and potentially replace conventional cancer treatments.

## Figures and Tables

**Table 1:** Studies of bacteriocins towards different cancer cell lines using different assays.

Bacteriocin	Origin Bacteria	Type of Cancer	Type of Cell Line	Effect	Type of Assay	References
Nisin	*Lactococcus lactis*	Head and neck cancer	HNSCC cells	Reduced tumour volume in mice model by about 50% using dosage of 200 mg/kg	Measurement of tumour volume using mice tumour model	[38]
		Colorectal cancer	LS180, SW780, HT29 and Caco-2 colorectal cancer cells	Reduced cell proliferation of LS180 (IC_50_ = 80–400 IU/mL), SW48, HT29 and Caco-2 (IC_50_ = 350–800 IU/mL)	MTT assay and trypan blue exclusion assay	[2]
		Breast cancer and liver cancer	MCF-7 human breast adenocarcinoma, HepG2 carcinoma cells	Inhibited cell proliferation of MCF-7 cell (IC_50_ = 105.46 μM) HepG2 cell (IC_50_ = 112.25 μM)	MTT assay and cell morphology analysis using an inverted optical microscope	[47]
Enterocin	*Enterococcus* sp.	Liver cancer	HepG2 carcinoma cell	Inhibited cell proliferation of HepG2 cell (IC_50_ = 15.643 μM)	Neutral red assay	[10]
Plantaricin	*Lactobacillus plantarum*	Colorectal cancer	E705 colon cancer cells	Inhibitory effect of cell proliferation of nearly 30% at 10 ng/mL	MTT assay	[48]
Pediocin	*Pediococcus acidilactici*	Colorectal cancer and cervical cancer	HT29 colon adenocarcinoma, HeLa cervical adenocarcinoma cells	Inhibited the growth of HT29 cell (Undialysed: 55.0 ± 4.8%, Dialysed: 53.7 ± 7.0%) HeLa cell (Undialysed: 52.3 ± 6.0%, Dialysed: 15.6 ± 4.0%)	MTT assay	[49]
Bovicin	*Streptococcus bovis*	Breast cancer and liver cancer	MCF-7 human breast adenocarcinoma, HepG2 carcinoma cells	Inhibited cell proliferation of MCF-7 cell (IC_50_ = 279.39 μM) HepG2 cell (IC_50_ = 289.30 μM)	MTT assay and cell morphology analysis using an inverted optical microscope	[48]
Microcins	*Klebsiella pneumoniae*	Colorectal cancer	HT29 and SW620 colorectal adenocarcinoma cell lines	Decreased in cancer cell viability HT29 cell (treatment with 60 μg/mL reduces growth up to 50%) SW620 cell (treatment with 60 μg/mL reduces growth up to 69%) Significant reduction of SW620 tumour size	Flow cytometry and measurement of tumour size	[50]
Others	*Lactococcus garvieae*	Colorectal cancer	HT29 colon adenocarcinoma cells	Induced cell death at a low dosage of 2 μg/mL. Apoptosis of cancer cells observed through DAPI staining	MTT assay and DAPI staining	[51]
	*Bacillus amyloliquefaciens*	Lung cancer	A549 human alveolar epithelial cell line	The proliferation rate of less than 50% from 40 μg/mL to 200 μg/mL after 72 h of incubation	MTT assay, morphology analysis using a fluorescent microscope	[52]
	*Lactobacillus delbrueckii*	Cervical cancer, breast cancer, fibrosarcoma, lung cancer	HeLa cervical adenocarcinoma cells, MCF-7 human breast adenocarcinoma, HT1080 human fibrosarcoma cell line, H1299 non-small lung carcinoma	Cytotoxicity at 10 μM, MCF-7 = 60% cytotoxicity HT1080 and H1299 = 40% cytotoxicity HeLa = no significant Cytotoxicity at 10 μM, All cell line = 50% cytotoxicity	Trypan blue exclusion assay	[53]
